# Impact of diabetes mellitus on clinical outcomes in patients affected by Covid-19

**DOI:** 10.1186/s12933-020-01047-y

**Published:** 2020-06-11

**Authors:** Celestino Sardu, Giuseppe Gargiulo, Giovanni Esposito, Giuseppe Paolisso, Raffaele Marfella

**Affiliations:** 1 Department of Advanced Medical and Surgical Sciences (DAMSS), University of Campania “Luigi Vanvitelli”, Piazza Miraglia, 2, 80138 Naples, Italy; 2 Division of Cardiology, Department of Advanced Biomedical Sciences, University Federico II of Naples, Via S. Pansini 5, 80131 Naples, Italy

**Keywords:** Type 2 diabetes mellitus, Covid-19, Micro-vascular disease, Several disease

## Abstract

A possible association could exist between type 2 diabetes mellitus (T2DM) and Coronavirus-19 (Covid-19) infection. Indeed, patients with T2DM show high prevalence, severity of disease and mortality during Covid-19 infection. However, the rates of severe disease are significantly higher in patients with diabetes compared with non-diabetes (34.6% vs. 14.2%; p < 0.001). Similarly, T2DM patients have higher rates of need for Intensive Care Unit (ICU, 37.0% vs. 26.7%; p = 0.028). Thus, about the pneumonia of Covid-19, we might speculate that the complicated alveolar-capillary network of lungs could be targeted by T2DM micro-vascular damage. Therefore, T2DM patients frequently report respiratory symptoms and are at increased risk of several pulmonary diseases. In addition, pro-inflammatory pathways as that involving interleukin 6 (IL-6), could be a severity predictor of lung diseases. Therefore, it looks intuitive to speculate that this condition could explain the growing trend of cases, hospitalization and mortality for patients with T2DM during Covid-19 infection. To date, an ongoing experimental therapy with monoclonal antibody against the IL-6 receptor in Italy seems to have beneficial effects on severe lung disease and prognosis in patients with Covid-19 infection. Therefore, should patients with T2DM be treated with more attention to glycemic control and monoclonal antibody against the IL-6 receptor during the Covid-19 infection?

In last months we are seeing the higher spreading of coronavirus-19 (Covid-19) infectious disease [1–4]. Notably, Covid-19 infection is showing us the characteristics of a pandemic disease, with thousands of cases, and higher rate of hospital admissions and deaths [1–4]. In this setting, from recent published larger trials, we could say that type 2 diabetes mellitus (T2DM) appears as a frequent co-morbidity of Covid-19 infectious disease [1–4]. To date, as first the diagnosis of T2DM is present in larger proportion of patients admitted to hospitals; secondly, T2DM is one of main cause of death in patients with Covid-19 [1–4]. Indeed, analyzing these data, the patients with T2DM have high prevalence, severity of disease and mortality during Covid-19 infection [1–4]. These features of pandemic Covid-19 infection were more evident comparing the proportion of severity endpoints among main recent studies, between diabetic and non-diabetic patients (Fig. 1). Indeed, in the large cohort by Guan et al. the 15.7% of patients presented with severe disease, but the rates of severe disease were significantly higher in patients with diabetes compared with non-diabetes (34.6% vs. 14.2%; p < 0.001) [1]. Similarly, 6.1% of patients experienced the composite endpoint, that was again significantly higher among diabetic vs. non-diabetics patients (22.2% vs. 4.8%; p < 0.001) [1]. Thus, when pooling data from 3 other studies, patients with diabetes have higher rates of need for Intensive Care Unit (ICU, 37.0% vs. 26.7%; p = 0.028) [2–4], (Fig. 1). However, assuming that patients with T2DM represent a high proportion of patients with Covid-19 and of patients with worse prognosis, we have to raise few questions. As first, we would elucidate the possible pathogenic mechanisms linking T2DM to Covid-19 pneumonia. Secondly, we would like to address the mechanisms causing worse prognosis in T2DM patients with Covid-19. Thus, we could speculate about the possible best therapies for T2DM patients with Covid-19.

**Fig. 1:**
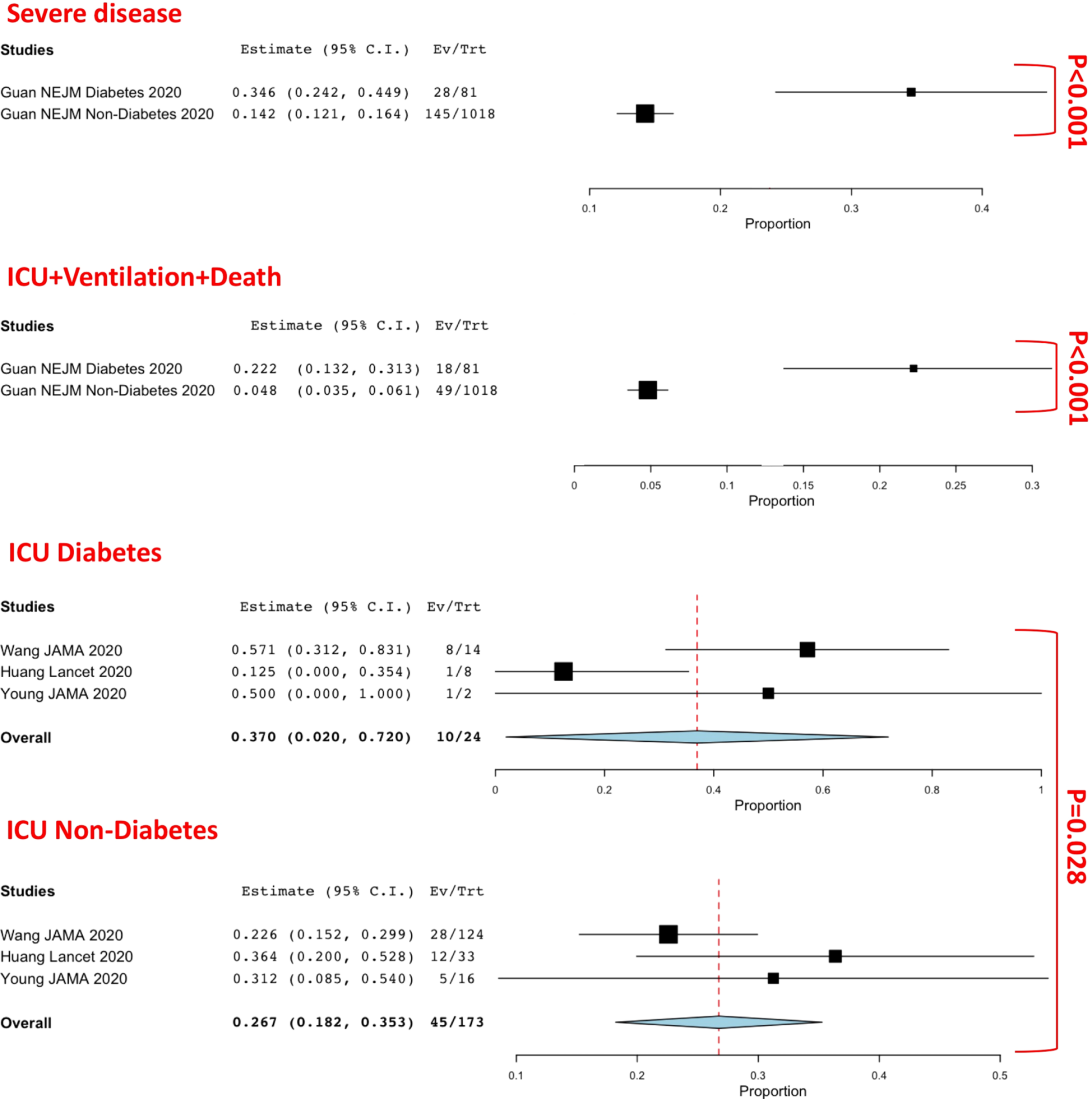
Proportion of severity endpoints among main recent studies. We performed a broad literature research in PubMed up to March 12, 2020. All articles dealing with coronavirus disease 2019 were screened. Main studies reporting characteristics and outcomes of patients affected by coronavirus disease with data referring to those with or without diabetes were extracted. Summary proportion estimates of the outcomes of interest were pooled, using inverse-variance weights obtained from a random-effects meta-analysis with 95% confidence interval. Statistical significance was set at p < 0.05. All analyses were performed in Open Meta-Analyst. In the study by Young et al., data stratified by diabetes referred to patients requiring or not requiring supplemental O2, which included but was not restricted to Intensive Care Unit (ICU)

Regarding the pathogenesis of T2DM and Covid-19 pneumonia, we have to deeply analyze the pathogenic mechanisms of lung disease in patients with T2DM [5]. The lung has a complicated alveolar-capillary network, which may be targeted by T2DM [5]. Indeed, T2DM causes a micro-vascular damage in patients with lung disease [5]. However, patients with T2DM frequently report respiratory symptoms and are at increased risk of several pulmonary diseases [5]. Looking about the molecular mechanisms implied in micro-vascular damage in patients with T2DM, we have to mention the over inflammation [5]. Indeed, in patients with T2DM the insulin resistance and altered glucose homeostasis lead to alveolar capillary micro-angiopathy and interstitial fibrosis via over-inflammation [5]. Several molecular mechanisms, which are induced by over-inflammation, have been suggested to explain the micro-vascular disease, and the consequent endothelial dysfunction and damage in lungs of patients with T2DM. One such mechanism of these pro-inflammatory endothelial pathways of small vessels is the interleukin 6 (IL-6). IL-6 is a well-known biomarker of inflammation and metabolic dysfunction, and it has been suggested as a severity predictor in lung diseases [5].

Specifically, T2DM patients, as compared to non-T2DM subjects, present with significantly higher plasma levels of IL-6 [6, 7] accompanied by augmentation of other pro-inflammatory cytokines like interleukin 18 (IL-18) [6] and metalloproteinase 12 (MMP-12) [7]. To date, in T2DM patients the chronic and systemic inflammation is associated with abnormal clot formation [6]. Indeed, T2DM sample’s coagulation profiles are significantly more hyper-coagulable when compared to healthy samples [6]. Thus, deregulated inflammatory circulating molecules may in part be responsible for a hyper coagulable state and vascular dysfunction in the T2DM patients [6]. Intriguingly, in T2DM patients the higher levels of interleukins and MMP-12, are independently related to several structural and functional markers of preclinical cardiovascular organ damage [7].

Moreover, targeting IL-6, a key molecule within the inflammatory cytokine network, may be a novel therapeutic strategy for COVID-19-induced cytokine release syndrome [8]. Indeed, IL-6 could be differently expressed among ICU vs. non-ICU patients, and it could be evaluated for risk stratification and therapeutic effect monitoring by IL-6 blockade biological agents [8].

Notably, the progression of Covid-19 pneumonia could cause thromboembolic events and reduction of lung functionality [1–4]. All these events are mainly seen in patients with T2DM and are manifestations of micro vascular endothelial dysfunction and damage [5]. To date, in patients with T2DM these effects could cause both restrictive and obstructive lung function impairment, including reduction in forced expiratory volume in 1 s, forced vital capacity, lung diffusing capacity and lung elastic recoil [5].

Therefore, it looks intuitive to speculate that this pathogenic condition could explain the growing trend of cases, hospitalization and mortality for patients with T2DM during Covid-19 infection [1–4]. Furthermore, the lung disease in T2DM patients looks to be linked to both hyperglycemia and IL-6 pathways [5]. Notably, to support these concepts, an ongoing and promising experimental therapy with monoclonal antibody against the IL-6 receptor in Italy seems to have beneficial effects on severe lung disease and prognosis in patients with Covid-19 infection. Indeed, the monoclonal antibody against IL-6 tocilizumab could reduce IL-6 levels, and is an effective treatment option in Covid-19 patients with a risk of cytokine storms [8, 9]. Precisely, repeated doses of the monoclonal antibody against IL-6 tocilizumab have been recommended for critically ill patients with elevated levels of this cytokine [8]. Indeed, although tocilizumab reduces inflammation in all critically ill patients rapidly, it could be not effective at single dose [9]. Therefore, in few critically ill patients this could cause a persistent and dramatic increase of IL-6 with a clinical outcome of disease aggravation [9]. Thus, for critically ill patients with elevated IL-6, authors recommend the repeated dose of the tocilizumab [9].

Therefore, the observation of all these complex data could raise few questions. The first question is: can we do more for T2DM patients with Covid-19? Thus, a more accurate and precise risk stratification for patients with T2DM and Covid-19 infection is needed. However, we might speculate that, classifying patients with T2DM as high-risk population during Covid-19 infection, we could immediately start more aggressive therapies to control and reduce systemic and lung inflammation. This could cause the reduction of lung disease progression, and to prevent worse prognosis in patients with T2DM.

The second question, but not less relevant is: should patients with T2DM be particularly treated with more attention to glycemic control and monoclonal antibody against the IL-6 receptor during the Covid-19 infection? Thus, we might speculate that, a more intensive therapy with insulin infusion to target a tight glycemic control could be necessary to ameliorate glucose homeostasis and clinical outcomes in patients with T2DM and Covid-19 infection. Parallelly, the better glycemic control could cause amelioration of insulin resistance and of patients’ response to Covid-19. Finally, we cannot exclude that this could augment the therapeutic response to the anti IL-6 therapies. On other hand, these hypotheses are speculative and need to be clarified in further ongoing trials on T2DM and Covid-19.

## Data Availability

All data and materials used are available.

## Abbreviations

T2DM: Type 2 diabetes mellitus
Covid-19: Coronavirus-19
ICU: Intensive Care Unit
IL-6: Interleukin 6
IL-18: Interleukin 18
MMP: Metalloproteinases
